# Soil Properties and Maize Yield Improvement with Biochar-Enriched Poultry Litter-Based Fertilizer

**DOI:** 10.3390/ma15249003

**Published:** 2022-12-16

**Authors:** Jiri Holatko, Tereza Hammerschmiedt, Jiri Kucerik, Tivadar Baltazar, Maja Radziemska, Zdenek Havlicek, Antonin Kintl, Iwona Jaskulska, Ondrej Malicek, Martin Brtnicky

**Affiliations:** 1 Department of Agrochemistry, Soil Science, Microbiology and Plant Nutrition, Faculty of AgriSciences, Mendel University in Brno, Zemedelska 1, 613 00 Brno, Czech Republic; 2 Agrovyzkum Rapotin, Ltd., Vyzkumniku 267, 788 13 Rapotin, Czech Republic; 3 Faculty of Chemistry, Institute of Chemistry and Technology of Environmental Protection, Brno University of Technology, Purkynova 118, 612 00 Brno, Czech Republic; 4 Institute of Environmental Engineering, Warsaw University of Life Sciences, 02-787 Warsaw, Poland; 5 Department of Animal Morphology, Physiology and Genetics, Faculty of AgriSciences, Mendel University in Brno, Zemedelska 1, 613 00 Brno, Czech Republic; 6 Agricultural Research, Ltd., Zahradni 1, 664 41 Troubsko, Czech Republic; 7 Faculty of Agriculture and Biotechnology Bydgoszcz, Bydgoszcz University of Science and Technology, 85-796 Bydgoszcz, Poland

**Keywords:** co-matured organic amendment, pyrolyzed nutrient stabilizer, soil respiration enhancement, crop yield improvement

## Abstract

Conversion of poultry litter into fertilizer presents an environmentally friendly way for its disposal. The amendment of stabilizing sorption materials (e.g., biochar) to broiler chicken rearing seems promising, as it protects produced litter from nutrient losses and improves fertilizing efficacy. Thus, a pot experiment was carried out with maize and organic fertilizers produced from biochar-amended chicken bedding. The properties of three types of poultry-matured litter, amended with biochar at 0%, 10% and 20% dose, were analyzed. These matured litters were added to soil and physicochemical, biological properties and dry aboveground crop biomass yield were determined. Both biochar doses improved matured litter dry matter (+29%, +68% compared to unamended litter) and organic carbon (+5%, +9%). All three fertilizers significantly increased dry plant aboveground biomass yield (+3% and +42% compared to control litter-treated variant) and N-acetyl-β-D-glucosaminidase activity (+51%, +57%) compared to unamended control soil. The 20% biochar poultry-matured litter derived the highest dry plant aboveground biomass, highest respiration induced by D-glucose (+53%) and D-mannose (+35%, compared to control litter-treated variant), and decreased pH (−6% compared to unamended control). Biochar-derived modification of poultry litter maturation process led to organic fertilizer which enhanced degradation of soil organic matter in the subsequently amended soil. Furthermore, this type of fertilizer, compared to conventional unamended litter-based type, increased microbial activity, nutrient availability, and biomass yield of maize in selected biochar doses, even under conditions of significant soil acidification.

## 1. Introduction

Poultry production is currently expanding and intensifying, and relentless discharge of manure into the environment could become a serious source of global pollution [1]. Similar to waste treatment technologies from other types of livestock production (cattle, pig breeding), aerobic composting has become important for the efficient decomposition of organic matter and nutrient transformation, reduction and detoxification of waste stocks and maximizing the efficiency of the use of production resources contained in poultry manure, usable as organic fertilizer in soil [2,3,4].

However, greenhouse gas emissions and nitrogen loss [5,6] are major drawbacks of composting, leading to a reduction in the agricultural value of the final manure product and the concomitant release of odorous gases are harmful to human health and the environment [7,8]. Therefore, there is an increasing motivation to control the quality of the end product of poultry litter composting with the intention of reducing greenhouse gas and ammonia emissions [9], reduce nitrogen loss, deodorize [10], speed up the composting process [11], efficiently convert nutrients, and decompose organic matter, along with its preservation (e.g., degree of humification) [8,12]. The most preferred approach is the amendment of various stabilizing sorption materials such as biochar, mineral clays like zeolite and bentonite [13,14,15]. These approaches are based either on the addition of an adsorbent supplement to the litter during poultry breeding [16,17] or during poultry litter processing, i.e., co-composting [18,19,20,21,22,23] or co-application to soil [24,25,26,27].

Enrichment of poultry manure with biochar has been found to show mostly positive effects on ammonia emissions [10,11,28], nitrogen mineralization [29,30] and retention [10], organic matter degradation and humification [29,31,32,33], microbial community composition and succession [10,34,35], plant growth and crop yield [24,25,27]. It was also found that a greenhouse experiment with simultaneous application of poultry litter and biochar as a nutrient immobilizer to soil planted with bermudagrass (*Cynodon dactylon* resulted in a 25, 24, 30, 29 and 35% reduction in C, N, P, Cu and Zn losses from the soil, respectively [16]. Another study [36] found that the addition of biochar to the poultry litter gave the poultry litter different characteristics to the resulting fermented manure; the addition of 20% biochar showed reduced moisture, increased pH, higher peaks of CO_2_ and temperatures during composting compared to 5% biochar. The decomposition of poultry litter was accelerated when amended with biochar, but there was no difference in its weight loss when biochar was added. Biochar absorbed NH_3_ and water-soluble NH_4_^+^, thereby reducing ammonium nitrogen losses from manure by up to 64% [36].

The aim of this study was to evaluate the effect of biochar (which was added in two doses (10 and 20% *w*/*w*) to broiler litter) on the quality of the resulting products used as organic fertilizers. The novelty of this research lies in the original approach of combining amendment of litter with biochar during experimental broiler chicken rearing and long-term maturation under low-temperature conditions in paper bags (4 months at 14 ± 4 °C). This innovative approach could decrease emissions of CO_2_, NH_3_, CH_4_, and other gases, and reduce losses of nitrogen and other nutrient elements thought volatilization. The matured litter fertilizers were tested for their effect on the physico-chemical and biological properties of soil, and plant biomass yield in a pot experiment. It was hypothesized that: (I.) Biochar in poultry litter provides nutrient stabilization (from volatilization) during transformation and mineralization, resulting in a nutrient richer product, which derives higher soil enzyme activity values and higher soil respiration rates after its application to soil. (II.) Enriched matured litter-derived reduction of nitrogen losses and increasing mineralization of other nutrients in amended soil promotes higher fertilization of maize and higher plant biomass yields. The details of the experimental design and technical procedures used for determination of soil properties and plant biomass yield are described in Methods and Materials sections; obtained findings and measured values are presented in the Results section, which is followed by the Discussion section, which comments, explains, and compares the findings with up-to-date knowledge of the research issue. A final summary is included in the Conclusions.

## 2. Materials and Methods

### 2.1. Poultry Matured Litter Production and Analyses

Biochar was incorporated into the litter in doses of 0%, 10% and 20% during an experimental broiler chicken rearing period of 31 days. In the experiment, commercial biochar (Sonnenerde GmbH, Riedlingsdorf, Austria) produced from agricultural waste (cereal bran and chaff, sunflower hulls, fruit peels and pulp) at 600 °C was used. The basic properties were: C 87%, C:N 289, pH 8.5, BET specific surface 289 m^2^·g^−1^. Fresh litter was thoroughly mixed and left to mature for 4 months at 14 ± 4°C. Afterwards, the final maturated litter was mixed again, and three samples of each treatment were taken to determine the basic properties. Dry matter content (DM) was measured gravimetrically according to [37], ash according to [38], carbon and organic carbon (C_org_) was established according to [39] using the wet oxidation of chromic acid. Available P was determined according to [40], extracted using the Mehlich III reagent [41] and then analyzed using atomic emission spectroscopy (The Agilent 55B AA, Agilent, CA, USA). Total Kjeldahl nitrogen (N) was determined according to (ISO 11261:1995) [42].

### 2.2. Pot Experiment

The experimental soil was prepared by mixing with silty clay loam (USDA Textural Triangle) Haplic Luvisol (WRB soil classification) collected in a field near the town Troubsko (Czech Republic) from a depth 0–15 cm, and a fine quartz sand (0.1–1.0 mm; ≥95% SiO_2_) in a 1:1 weight ratio. Some 30 g of matured poultry litter (equal to 10 t∙ha^−1^) was mixed with the 5 kg of experimental soil. The control treatment contained only 5 kg of soil. Each treatment was prepared in five replications. The following treatments were tested: (unamended) control, poultry litter (M), poultry litter + 10% biochar (M + B10), poultry litter + 20% biochar (M + B20). All pots were left in the greenhouse under the following day/night conditions: 20/12 °C, 45/60% air relative humidity, 12-h photoperiod, soil moisture was maintained at 60% of water holding capacity. After 6 weeks of incubation, all pots were sown with seven maize seeds. After 2 weeks the plants were reduced to the two most robust in each pot. Maize was grown for 12 weeks under the above conditions. Then the plants were cut at the ground level and the aboveground biomass (AGB) was dried at 60 °C and weighed on laboratory scales. The pots were re-sown with maize again followed by the same 12-week growing period. At the end of the experiment, plants were cut at ground level and AGB was dried at 60 °C, weighed on laboratory scales and cumulative dry aboveground biomass (AGB dry) was calculated. Moreover, a mixed soil sample was taken from each pot.

### 2.3. Soil Analyses

Soil samples were sieved through a 2 mm mesh sieve. Air-dried samples were used for pH analysis in CaCl_2_ (ISO 10390:2005) [43]. Freeze-dried samples were tested for enzymatic activities: N-acetyl-β-D-glucosaminidase (NAG), phosphatase (Phos), arylsulfatase (ARS), and β-glucosidase (GLU) were determined spectrophotometrically using 4-nitrophenyl derivates of specific substrates (ISO 20130:2018) [44]. The samples stored at 4 °C were used for measurement of substrate-induced respirations (SIR) (D-glucose (Glc-SIR), D-trehalose (Tre-SIR), D-mannose (Man-SIR) (Campbell et al. 2003) [45]) using the MicroResp^®^ device (The James Hutton Institute, Dundee, UK) and spectrophotometric measurement of chromogenic indicator of CO_2_ emission.

### 2.4. Soil Analyses

All statistical analyses were carried out using freely available program R, version 3.6.1. [46]. For characterization of the relationship among soil properties with dependence of selected treatments, a principal component analysis (PCA) was performed. For testing the statistical effect of a selected treatment to the soil properties, a one-way analysis of variance (ANOVA) type I (sequential) sum of squares was used. For detecting the statistically significant difference after ANOVA the Tukey’s honest significant difference (HSD) test at significance level 0.05 was employed. Factor level means were determined with a treatment contrast. Model checking was performed with the help of different diagnostic plots. In addition, a Shapiro-Wilk test for the verification of normality and the Levene’s test for the verification of homogeneity of variances were also performed at a significance level of 0.05. The Pearson correlation coefficient was used to determine the linear correlation among soil properties.

## 3. Results

### 3.1. Poultry Matured Litter Properties

**Figure 1:**
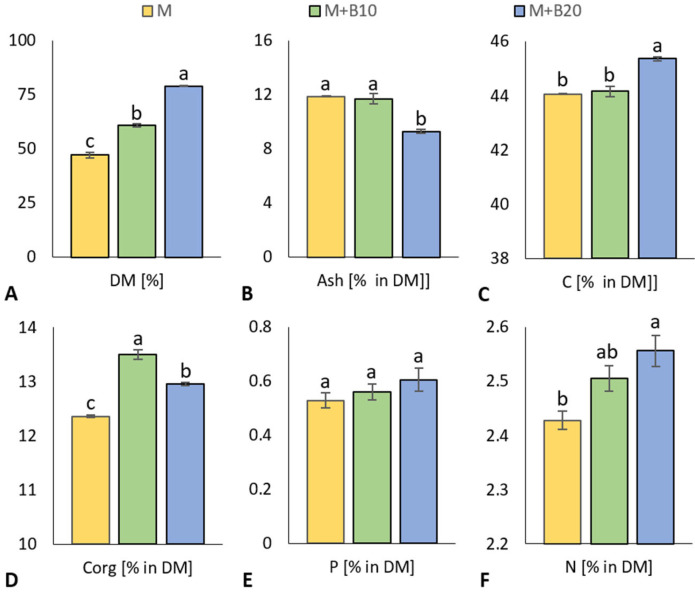
Dry matter (**A**), ash (**B**), total (**C**) and organic carbon (**D**), phosphorus (**E**), total nitrogen (**F**) in poultry litter treatments produced from untreated and biochar (10 and 20% *w*/*w*)-enriched litter. Displayed are mean values with error bars (standard error of mean); letters indicate differences between values at the statistical level of significance *p* ≤ 0.05.

Due to the different dose of biochar added to poultry litter (0, 10, 20% *w*/*w*), all three types of produced matured litter differed significantly in dry matter (DM) values, which increased in direct proportion to the added weight up to the highest M + B20 values, Figure 1A. Despite having the highest DM content, M + B20 had a significantly lower ash content compared to the other two litters M and M + B10, Figure 1B. Concurrently, higher access of biochar-derived carbon in M + B20 litter caused a significant increase in the total carbon (C), as compared to other treatments, Figure 1C. Organic carbon (C_org_) was significantly increased in both biochar-enriched treatments (M + B10, M + B20) in comparison to the unamended litter (M); however, M + B10 exerted significantly higher C_org_ compared to M + B20, Figure 1D. In contrast, no significant differences in pH values (M 6.56, M + B10 6.88, M + B20 7.08, in average) were observed among all litter treatments, although the pH in water of the added biochar was alkaline (pH 9.8) and was expected to affect the final alkalinity of the produced litter. The average phosphorus content increased with increasing doses of added biochar, but the differences were statistically insignificant, Figure 1E. The N sequestration and stabilization associated with biochar was also responsible for the significantly increased total nitrogen (N) content of M + B20 litter compared to the control litter M, although M + B10 showed only an insignificant difference, Figure 1F.

### 3.2. Soil Properties

**Figure 2:**
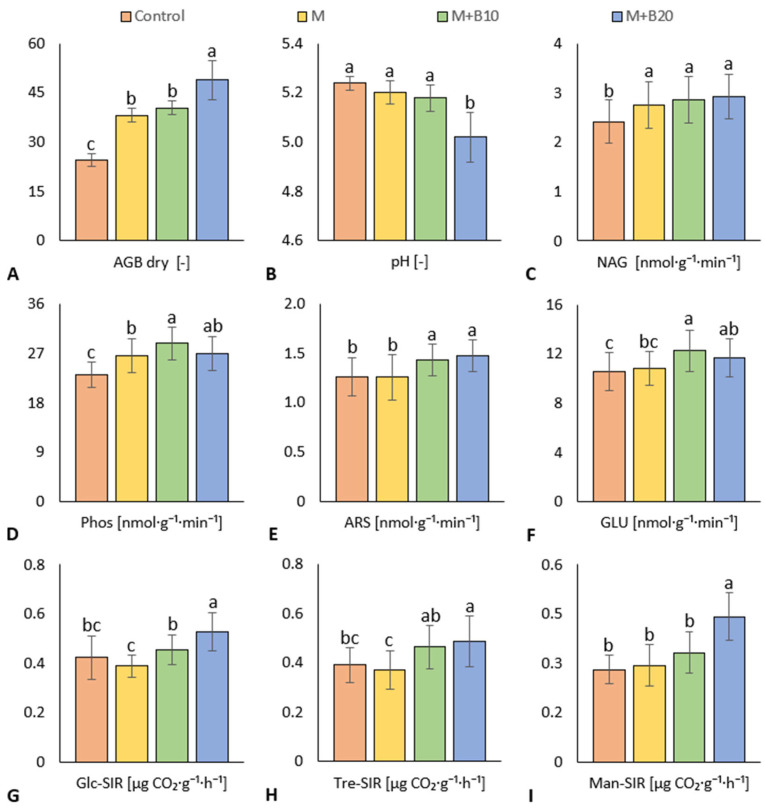
Dry aboveground biomass (**A**), soil pH (**B**), N-acetyl-β-D-glucosaminidase (**C**), phosphatase (**D**), arylsulfatase (**E**), β-glucosidase (**F**), D-glucose-induced soil respiration (**G**), D-trehalose- induced soil respiration (**H**) and D-mannose-induced respiration (**I**) in treatments amended with poultry litter produced from untreated and biochar (10 and 20% *w*/*w*)-enriched litter. Displayed are mean values with error bars (standard error of mean); letters indicate differences between values at the statistical level of significance *p* ≤ 0.05.

Dry aboveground biomass (AGB dry) was significantly increased in all litter-amended treatments in comparison to the control, Figure 2A. Litter enriched with a lower dose of biochar (M + B10) did not cause significantly higher AGB dry compared to untreated litter, while litter with a higher dose of biochar (M + B20) did and moreover achieved a significantly higher aboveground biomass yield. On the contrary, a significant decrease in soil pH was found for the M + B20 treatment as compared to other treatments, Figure 2B.

Soil enzymes were determined as indicators of nutrient transformation and mineralization activities. N-acetyl-β-D-glucosaminidase (NAG), an indicator of the amount and rate of decomposition of fungal biomass in soil, was significantly increased in both biochar-enriched litter treatments (M + B10, M + B20) compared to the control, Figure 2C. Lower soil pH is advantageous for fungal growth, but the most acidic treatment (M + B20) showed no significant difference in NAG compared to the unamended litter treatment (M). All litter-amended variants (M, M + B10, M + B20) also showed increased phosphatase (Phos) activity compared to the unfertilized control soil, Figure 2D. On the contrary, arylsulfatase activity (ARS) was significantly higher in both biochar-enriched treatments (M+B10, M+B20) as compared to untreated litter and control, Figure 2E. β-glucosidase (GLU) was significantly the highest in M+B10 soil, Figure 2F.

D-glucose-induced soil respiration (Glc-SIR) was measured as an indicator of soil potential to carbon (C) mineralization. In general, increased access of carbon in higher biochar-amended treatment caused stimulation of C mineralization. Therefore, the M+B20 treatment showed significantly the highest Glc-SIR, Figure 2G. In order to determine the preference of soil microbial community in C substrate utilization, D-trehalose (Tre-SIR)-and D-mannose (Man-SIR)-induced respiration were measured. Tre-SIR and Man-SIR were both significantly increased in M+B20 in comparison to the control and M, Figure 2H–I. Moreover, M+B10 exerted significantly higher Tre-SIR compared to M treatment, Figure 2H.

## 4. Discussion

### 4.1. Poultry Matured Litter Properties

As expected, the M + B20 litter showed the highest DM compared to the other two litters (Figure 1A), but a significantly lower ash content (Figure 1B), which was caused by high biochar content. Sludge and livestock manure are known to have higher ash content and lower organic constituents compared to plant biomass [47], which was the pyrolysis feedstock for the production of the biochar used in this experiment. The highest total carbon content of litter in treatment M + B20 (Figure 1C) was also predictable as this treatment received the highest rate of added biochar with significantly higher carbon content than poultry litter. Biochar-enriched variants (M + B10, M + B20) contained more organic carbon (C_org_) than unenriched litter (Figure 1D). However, lower biochar dose promoted degradation of complex and recalcitrant biochar-derived compounds to more labile carbon in M + B10 as compared to M + B20. On the contrary, higher biochar-coupled sorption of organic matter in this variant mitigated microbial processes contributing to better carbon degradability. This is in the line with reduction in C_org_ stock in high biochar dose-treated organic fertilizer due to the sorption of carbon derived from the litter onto the biochar [48].

However, the significant effect of biochar on nutrient sorption and stabilization expectedly led to a significantly increased total nitrogen content in the M + B20 litter compared to the control litter M, Figure 1F. This finding was consistent with other studies that reported an increase in total nitrogen when litter and biochar were co-matured [12,24,31]. This beneficial effect on stabilizing nitrogen in modified manure through biochar-mediated mitigation of volatilization or other loss pathways was already reported too [20,28]. Poultry litter is known to be a rich source of phosphorus with a high phosphorus to nitrogen ratio, but its availability is reduced at an excessively low pH. The previously described positive effect of biochar on phosphorus utilization and availability in both litter and soil was predicted and in consent with other reports [12,49]. Nevertheless, there was no significant difference in phosphorus content among all litter variants, Figure 1E.

### 4.2. Soil Properties

Poultry litter is described as a beneficial agriculture fertilizer which is an effective source of nutrients, especially nitrogen and phosphorus [50,51]. The positive effect of poultry litter fertilization led to an increase in AGB dry in all fertilized variants compared to the control, but the highest yield was achieved by the M + B20 treatment, Figure 2A. This indicates an increased efficiency of fertilization with product prepared from poultry litter enriched with high doses of biochar. Biochar is generally known for its benefit to soil carbon and other nutrient element sequestration [52]. These results are in the line with the findings of several studies [51,53]. A putative reason was that the biochar amendment stimulated the mineralization of nutrients (nitrogen, phosphorus, sulfur, etc.), leading to decreased losses by leaching or volatilization [28], and accelerated transformation into the plant-available form. Sufficient access of these elements (namely P) for plant nutrition is globally important for the referred instant deficit in the agriculture systems worldwide [54]. Biochar immobilized volatile forms of sulfur (e.g., H_2_S) during the litter maturation and provided S-enriched soil organic matter to the respective soil variants. These presumptions were proven by the enhanced activities of Phos and ARS (Figure 2D–E), which enzymes were stimulated by application of fertilizer produced from biochar-enriched poultry litter. The significant increase in ARS and Phos activities in M+B10 soil compared to the control and M soil is consistent with references to the beneficial effect of poultry litter co-processed with biochar on soil nutrients availability to plants [24] and microbial nutrient conversion processes and enzyme activities [23,55]. The significant correlation (*p* ≤ 0.01) between AGB dry and Phos (r = 0.57) further confirmed the reported positive effect of co-matured biochar and poultry litter on Phos activity [23,35].

Yield of maize AGB dry was also affected by soil pH, which can be attributed to a significant negative correlation (*p* ≤ 0.001) between AGB dry and pH (r = −0.74), Figure A2. However, only treatment M+B20 showed significant decrease in soil pH, compared to other treatments. Some studies reported no effect or an alkalizing effect on soil pH from a high dose of biochar co-applied with poultry litter; nevertheless, it was at a lower biochar dose [12] or higher biochar dose together with much lower dose of poultry manure, i.e., strong excess of biochar [25]. On the contrary, soil amendment of biochar poultry litter fertilizer and pyroligneous solution significantly decreased the pH of saline soil [21]. Maize yields responded positively to an increasing dose of amended poultry litter, with a concomitant decrease in soil pH [56]. We speculate that the significant drop in pH observed in this work could be explained by increased microbiological activity in soil amended by M + B20. Due to its origin, poultry litter may contain more anaerobic microorganisms, which after the hydrolysis phase and the depletion of oxygen could initiate the formation of organic acids in the acidogenic phase.

**Figure A1:**
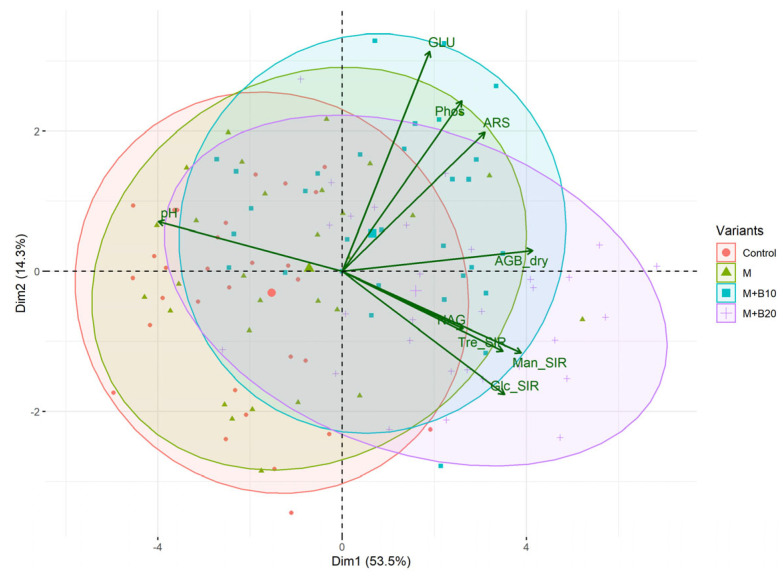
PCA biplot analysis of properties determined in the experiment.

**Figure A2:**
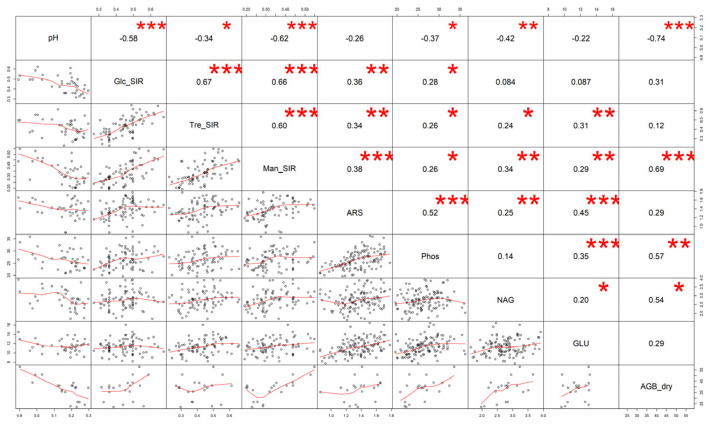
Pearson’s correlation matrix of properties determined in the experiment. The asterisks indicate a level of significance of statistical difference between the variables: * for *p* ≤ 0.05, ** for *p* ≤ 0.01, *** for *p* ≤ 0.001.

In addition to the hypothesized enhanced transformation of N, P, and S sources in biochar-enriched poultry litter-treated soil, there was an assumed and evidenced enhanced turnover of labile soil organic matter (SOM) pool in general, and increase in carbon mineralization enzymes in particular. Significantly increased GLU activity in M + B10 and M + B20 treatments proved the expected positive effect of biochar-enriched poultry litter on SOM turnover. These findings are consistent with reports of higher rates of SOM degradation when biochar and poultry litter were co-matured [15,29] or in matured litter-treated soils [57,58]. A PCA biplot also showed synergy of GLU, Phos, ARS, Figure A1. The values of substrate-induced respiration (SIR) indicated stimulated carbon mineralization potential; M + B20 exerted significantly increased Glc-, Tre-, Man-SIRs as compared to all other treatments (except of comparable Tre-SIR value in M + B10). Again, it was proven that poultry litter enriched with a higher dose of biochar positively affected microbial decomposition activity in the soil, resulting in an increased CO_2_ emission similar to that reported by other authors [53,59]. Glc-SIR correlated significantly (*p* ≤ 0.001) positively with Tre-SIR and Man-SIR (r were 0.67 and 0.66, respectively, Figure A2, corroborating the generally positive impact of poultry litter + biochar on aerobic degradation of organic matter, as previously reported [19,29]. Furthermore, Tre-SIR was considered as an indicator of fungal-associated activity together with NAG, which shows synergy on the PCA biplot, Figure A1. Since NAG activity, which monitors the turnover of fungal biomass in soil, was significantly increased in each of poultry litter-amended variant, these findings presumed an increased soil fungal abundance derived from all tested poultry litter treatments. This agrees with the reported increase in fungal biomass compared to bacterial biomass [23,60] and fungal diversity [35,61] during co-maturation of biochar + poultry manure, as well as in response to the respective manure addition to the soil [62]. These findings may indicate increased activity of microorganisms involved in the degradation of complex organic compounds such as hemicellulose (polymer which comprises of i.a. D-mannose) and fungal biomass (as D-trehalose is an abundantly secreted product of fungi) necromass. The increased ratio of fungi and bacteria was probably also related to the accelerated decomposition of structurally less degraded organic matter in poultry litter-amended soil, where retardation in hydrolysis could be caused by the lower temperature of the maturation process (14 ± 4 °C). It is known that fungi are mainly involved in the decomposition of complex organic compounds (e.g., cellulose, hemicellulose).

A synergy was observed on a PCA biplot, Figure A1, and a correlation on the Pearson’ matrix (*p* ≤ 0.001, r = 0.6) between Tre-SIR and Man-SIR, Figure A2. Moreover, the increased fungal biomass was probably favored by the low soil pH, as bacteria are much less sustainable in acidic soil than fungi [63]. Consequently, a negative correlation between fungal-related degradation indicators Tre-SIR (*p* ≤ 0.05, r = −0.34) and NAG (*p* ≤ 0.01, r = −0.42) with pH was revealed, Figure A2. Finally, acidity-mediated increase in fungal biomass turnover (NAG) and enhanced mineralization of plant-derived carbon (Man-SIR) putatively contributed to the higher maize aboveground biomass, as this was corroborated with a significant positive correlation of dry AGB with NAG (*p* ≤ 0.05, r = 0.54) and Man-SIR (*p* ≤ 0.001, r = 0.69), Figure A2.

## 5. Conclusions

The 10 or 20% addition in biochar dose to poultry litter and subsequent maturation significantly changed the properties of produced enriched litter in comparison to the control litter; dry matter and organic carbon content were higher for both biochar doses (DM +29%, +68% and C_org_ +5%, +9%, compared to unamended litter). A significant increase in total carbon and nitrogen content (C +3%, N +5%) and a decrease in ash content (Ash −22%) were found for the high (20%) biochar dose. These variably matured litters (with 10%, 20% BC), applied to soil, showed a positive impact on cumulative dry aboveground biomass yield (AGB dry +3% and +42% compared to the control litter-amended variant), probably due to the stimulation of fungal biomass and activity in soil which was indicated by the enhanced activity of N-acetyl-β-D-glucosaminidase (NAG +51% and +57% compared to unamended control). Both doses of biochar co-matured with poultry litter accelerated maturation and presumably increased the content of available nutrients as indicated by enhanced enzyme activities (arylsulfatase, phosphatase, β-glucosidase) and D-trehalose-induced respiration. Of the tested variants, the addition of poultry litter enriched with 20% of biochar had the highest benefit on soil health as it increased plant biomass yield, D-glucose (+53 compared to control litter-amended variant) and D-mannose (+35 compared to control litter-amended variant) induced respiration and led to the greatest decrease in pH (−6% compared to unamended control). The modification of organic fertilizer maturation in the amended soil conferred several desired properties to the product, e.g., increased degradation of organic matter, higher microbial activity, putatively improved nutrient availability, and higher maize biomass yield, even under conditions of significant soil acidification with selected biochar doses.

## Data Availability

All the data reported in this study was originally generated and can be requested from the corresponding author.

## Abbreviations

dry above-ground biomass	AGB dry
arylsulfatase	ARS
ash	Ash
biochar	BC
Brunauer–Emmett–Teller specific surface	BET
total carbon:nitrogen ratio	C:N)
litter carbon content	C
organic carbon	C_org_
dry matter	DM
D-glucose	Glc
β-glucosidase	GLU
D-mannose	Man
litter nitrogen content	N
N-acetyl-β-D-glucosaminidase	NAG
litter phosphorus content	P
phosphatase	Phos
*p*-value	*p*
Pearson’s correlation coefficient	r
soil organic matter	SOM
Substrate-induced respiration	SIR
total soil carbon	TC
total soil nitrogen	TN
D-trehalose	Tre
